# Case-control study: soft-tissue sarcomas and exposure to phenoxyacetic acids or chlorophenols.

**DOI:** 10.1038/bjc.1979.125

**Published:** 1979-06

**Authors:** L. Hardell, A. Sandström

## Abstract

In 1977 a number of patients with soft-tissue sarcomas and previous exposure to phenoxyacetic acids were described. Following from these observations a matched case-control study was made. Exposure to chlorophenols was also included in this study. The results showed that exposure to phenoxyacetic acids or chlorophenols gave an approximately 6-fold increase in the risk for this type of tumour. It was not possible to determine, however, whether the carcinogenic effect was exerted by these compounds or by impurities such as chlorinated dibenzodioxins and dibenzofurans that in almost all cases were part of the commercial preparations.


*Br. J. Cancer* (1979) **39**, 711

### CASE-CONTROL STUDY: SOFT-TISSUE SARCOMAS AND EXPOSURE TO PHENOXYACETIC ACIDS OR CHLOROPHENOLS

L. HARDELL AND A. SANDSTRÖM

*From the Center of Oncology, University Hospital, S-901 85 Umeå, Sweden*

Received 7 December 1978 Accepted 13 February 1979

**Summary.—In 1977 a number of patients with soft-tissue sarcomas and previous exposure to phenoxyacetic acids were described. Following from these observations a matched case-control study was made. Exposure to chlorophenols was also included in this study. The results showed that exposure to phenoxyacetic acids or chlorophenols gave an approximately 6-fold increase in the risk for this type of tumour. It was not possible to determine, however, whether the carcinogenic effect was exerted by these compounds or by impurities such as chlorinated dibenzodioxins and dibenzofurans that in almost all cases were part of the commercial preparations.**

IN the general debate on environmental hazards in Sweden, few topics have been discussed as vigorously as the phenoxyacetic acids. The debate has focused on their presumptive carcinogenic and teratogenic risks. There are no epidemiological or other reports that have firmly established a correlation between cancer and previous exposure to phenoxyacetic acids in human beings. In an investigation of Swedish railroad workers with exposure to different herbicides, a significantly higher tumour incidence and mortality rate was shown in those with exposure to amitrole, but initially the same findings did not apply to phenoxyacetic acids (Axelson & Sundell, 1974). In a re-analysis of this study doubts were raised about the phenoxyacetic acids as a specific group of products (Axelson & Sundell, 1977). In an accident at the BASF plant, Ludwigshafen, in 1953, 75 persons were exposed to trichlorophenol and chlorinated dioxins. Six malignant neoplasms causing death were found among those exposed, whereas only 3 were expected from comparison with general population data and 4 from comparison with an internal control group (Thiess & Frentzel-Beyme, 1978). Mice with peroral exposure to dioxin over 2 years showed an increased frequency of hepatomas over the control group (Tóth *et al.*, 1978). Another study on mice with peroral exposure to 2,4,5-trichlorophenoxyacetic acid (2,4,5-T) showed an increased tumour incidence in one of two studied strains (Muranyi-Kovacs *et al.*, 1976).

Recently a number of patients with soft-tissue sarcomas and previous heavy exposure to phenoxyacetic acids were reported (Hardell, 1977). This clinical observation resulted in a matched case-control study of this type of tumour. An analysis of exposure to chlorophenols was included in the investigation, as there are related processes in the production of phenoxyacetic acids and chlorophenols, and there may be similar impurities such as chlorinated dibenzodioxins and dibenzofurans in the commercial preparations.

Phenoxy herbicides have been used to control unwanted hardwoods in Swedish forestry since the beginning of the 1950s, usually as a mixture of 2,4-dichlorophenoxyacetic acid (2,4-D) and 2,4,5-T. In order to control mainly aspen, 2,4-D has been used in stump and basal-bark spraying. In farming, 4-chloro-2-methylphenoxyacetic acid (MCPA) has been the dominating herbicide.

Chlorophenols were also introduced in Sweden in the beginning of the 1950s.

About 90% of the consumption has been in sawmills as impregnates or to protect against blue stain in cut and newly sawn timber. Chlorophenols have been used as a fungicide for slime control in the production of paper pulp.

In addition these chemicals have been used as wood preservatives in paints and to waterproof leather and textiles. High levels of chlorinated dibenzodioxins, dibenzofurans and other impurities have been shown in the sawdust from sawmills, especially from the trimming-grading plant where the sawn timber is handled after chlorophenol treatment and drying (Levin *et al.*, 1976).

### MATERIALS AND METHODS

*Cases.*—The studied cases consisted of 21 living and 31 deceased male patients with soft-tissue sarcomas, ranging in age from 26 to 80 years and admitted to the Department of Oncology, University Hospital, Umeå during 1970–77.

*Controls.*—Four matched controls were selected for each case. For each living patient, 8 controls were extracted initially from the National Population Registry matched for sex, age and place of residence. Following a check that these controls were residents of the same municipality as the matched patient at the time of admission to the clinic, a criterion on which a total of 5 controls failed, the 4 ranging closest in age to the patient were used for the study. For dead patients, 10 controls were extracted initially from the National Registry for Causes of Death in order to match each of the deceased patients. They were matched for sex, age, year of death (for 1977, controls from 1976 were used) and place of residence. Deaths from malignant tumours or suicide were excluded. A variation of $\pm 5$ years from the age of the patient was accepted because of the small population in some municipalities. If this restriction was not fulfilled, complementary controls from a neighbouring, socio-economically similar municipality were extracted. This was the case for 9 municipalities or 29 controls in total. Dead controls might have been out of work for a long period of time before death and therefore have had less probability of exposure. For this reason their sick leaves and date of retirement were checked through the records of the Public Health Insurance Offices. A criterion was that the controls should have been working until 2 years before the retirement of the patient or, in the case of an unretired patient, until 2 years before his death. Because of this criterion 22 controls were excluded. In addition, one invalid, one person who could not be located and one person without relatives were excluded. As for the living patients, the 4 remaining controls closest in age to the patient were selected for the study.

*Assessment of exposure.*—The investigation started in January 1978. Those persons included in the investigations, for living patients and their controls the persons themselves, and for deceased patients and their controls the next of kin (defined in the following order: wife, children or parents, brother or sister), were if possible contacted by telephone in advance, without the specific nature of the investigation being disclosed. They then received by mail a questionnaire which consisted of a variety of questions about previous and present occupation, different kinds of exposure (especially chemical) in the working environment, smoking habits, etc. Special attention to phenoxyacetic acids or chlorophenols was thereby avoided. The answers were studied and supplemented over the phone by a person not associated with the department, and who did not know whether the interviewed persons were patients or controls. For deceased patients and controls the procedure was the same but in these cases contact was made with the next of kin.

To get as objective information as possible about exposure to phenoxyacetic acids, a questionnaire was also sent to the employers of the persons stating forest work, in order to verify their employment and the use of this chemical. Likewise a questionnaire was sent to sawmills and pulp industries about their use of chlorophenols in the production method.

Low levels of exposure (*i.e.* a maximum of one day) and late exposure, *i.e.* 5 years before the tumour was diagnosed, were not included for any of the 2 chemical groups. Sporadic use of impregnating agents during leisure was analysed separately.

*Statistical methods.*—Calculation of relative risk in the matched material was based on principles given by Miettinen (1970). The effect of matching for control of confounding factors was estimated as the quotient of the relative risk in the unmatched to the matched material (*cf.* Miettinen, 1972). The method described by Miettinen (1976) was used in the calculation of the confidence limits.

### RESULTS

The study included 260 persons: 52 patients and 208 controls. Thirty-one patients were deceased. The questionnaire was not answered by 2 controls (0·77%). Twenty-four (46%) of the patients lived in rural areas. According to the 1970 and 1975 census, 26·5% of the population in the age group 25–79 years in the admission region of the Department of Oncology lived in rural areas.

Exposure to phenoxyacetic acids or chlorophenols was registered in 36·5% of the patients and in 9·2% of the controls (Table I). The results obtained from the questionnaire sent to the employer about use of phenoxyacetic acids was uncertain and difficult to evaluate. Records of individual working manuals had not been kept, and the answers were mainly based upon reminiscence. Replies from the employers were obtained for 20/50 persons involved. As to the questionnaire about the use of chlorophenols in sawmills or pulp industry, the answer rate was 97%, and there was a good agreement with the statements given by the examined persons. This indicated that the questionnaires to patients and controls probably also gave reliable information about exposure to phenoxyacetic acids.

The relative risk for soft-tissue sarcomas after exposure to phenoxyacetic acids of chlorophenols was in the matched material 6·2, and after dissolving the matching 5·7 (95% confidence interval 2·9–11·3; Table II). To control the confounding factors the quotient between these relative risks was calculated as 5·7/6·2=0·9. This indicates that these confounding factors did not bias the result and the matching was therefore dissolved in the continued analysis.

TABLE 1.—*Exposure to phenoxyacetic acids (Ph) or chlorophenols (Ch) in the matched material (excluding 25 cases where the case and its 4 controls were all negative)*

| No. | Case | Control 1 | Control 2 | Control 3 | Control 4 |
|---|---|---|---|---|---|
| 01 | Ph | — | Ch | — | — |
| 02 | — | Ph | — | — | — |
| 10 | Ch | — | — | — | — |
| 11 | — | — | — | Ph | — |
| 12 | Ch | — | — | — | — |
| 16 | Ph | Ph | — | — | — |
| 18 | Ph | — | — | — | — |
| 19 | Ph | — | — | — | Ph |
| 20 | Ch | Ph | — | — | — |
| 22 | Ph | — | — | — | — |
| 23 | Ph | — | — | — | — |
| 25 | — | — | — | Ch | — |
| 27 | Ph | — | — | — | — |
| 33 | — | — | — | Ch | — |
| 34 | — | — | Ph+Ch | — | — |
| 35 | Ph | — | — | — | — |
| 39 | Ph+Ch | — | — | — | — |
| 43 | Ph | — | — | — | Ph |
| 47 | — | — | Ch | — | — |
| 49 | Ph | — | — | — | Ph |
| 50 | — | Ph | Ch | — | — |
| 51 | Ph | — | — | — | — |
| 52 | Ph | — | — | Ph | — |
| 54 | Ch | Ph | — | — | — |
| 56 | Ch | — | — | — | — |
| 57 | Ch | — | — | — | — |
| 58 | — | Ph | Ph | — | Ph |

TABLE II.—*Exposure to phenoxyacetic acids and/or chlorophenols in the total material*

| | Exp. | Non-exp. | Total |
|---|---|---|---|
| Case | 19 | 33 | 52 |
| Control | 19 | 187 | 206 |
| | 38 | 220 | 258 |

Relative risk: 5·7 (95% confidence interval 2·9–11·3).
$\chi^2 = 24{\cdot}6$.
$P < 0{\cdot}001$.

As there might be some doubt about the information obtained from relatives compared with that obtained at first hand, a subdivision of the patients and controls was made in the 2 groups of living and deceased. The relative risk for the 21 living patients and their controls was then calculated as 9·9, and for the 31 dead patients and their controls as 3·8. This indicated that second-hand information underestimated the risk.

### *Phenoxyacetic acids*

The effect of exposure to phenoxyacetic acids only was analysed as part of

TABLE III.—*Cases and controls exposed to phenoxyacetic acids. Duration of latency and exposure. Time of exposure within 5 years before the diagnosis has not been included*

| No. | Duration of latency (yrs) | Duration of exposure (months·days) | Chemical | Nature of work |
|---|---|---|---|---|
| Cases | | | | |
| 010 | 15–7 | 18·14 | 2,4,5-T | Work on wet ground |
| 160 | 19 | 00·07 | 2,4-D, 2,4,5-T | Supervisor |
| 180 | 15–13 | 00·07 | MCPA | Tractor-mounted spraying |
| 190 | 27–14 | 49·00 | 2,4-D, 2,4,5-T | Knapsack spraying |
| 220 | 15–8 | 08·00 | 2,4,5-T | Work on wet ground |
| 230 | 20–14 | 08·15 | 2,4-D, 2,4,5-T | Basal-bark spraying, knapsack spraying |
| 270 | 11–0 | 03·21 | 2,4-D, 2,4,5-T | Mistblowing |
| 350 | 18–5 | 05·03 | 2,4-D, 2,4,5-T | Knapsack spraying |
| 390 | 19–9 | 00·02 | 2,4-D (?) | Mistblowing |
| 430 | 20–18 | 03·00 | 2,4-D, 2,4,5-T | Truck-mounted spraying |
| 490 | 10–5 | 03·21 | 2,4-D, 2,4,5-T | Knapsack spraying |
| 510 | 9–3 | 05·00 | 2,4-D, 2,4,5-T | Knapsack spraying |
| 520 | 12–8 | 03·00 | 2,4-D, 2,4,5-T | Basal-bark spraying, knapsack spraying |
| Controls | | | | |
| 021 | 22 | 00·21 | Weedone | Truck-mounted spraying |
| 113 | 17 | 00·06 | 2,4-D, 2,4,5-T | Knapsack spraying |
| 161 | 19–10 | 03·21 | 2,4-D, 2,4,5-T | Knapsack spraying |
| 194 | 24–21 | 02·14 | 2,4-D, 2,4,5-T | Supervisor |
| 201 | 8–6 | 09·00 | 2,4,5-T | Basal-bark spraying, knapsack spraying |
| 342 | 6–0 | 00·05 | MCPA | Tractor-mounted spraying |
| 434 | 20–16 | 05·00 | 2,4-D | Basal-bark spraying |
| 494 | 18–15 | 01·00 | 2,4-D, 2,4,5-T | Knapsack spraying |
| 501 | 17–0 | 08·10 | MCPA, 2,4,5-T | Tractor-mounted spraying, knapsack spraying |
| 523 | 9–1 | 06·00 | 2,4-D, 2,4,5-T | Truck-mounted spraying, basal-bark spraying |
| 541 | 11–10 | 01·00 | 2,4-D, 2,4,5-T | Basal-bark spraying, knapsack spraying |
| 581 | 14–3 | 10·00 | 2,4-D, 2,4,5-T | Basal-bark spraying, knapsack spraying |
| 582 | 12–9 | 04·00 | 2,4-D, 2,4,5-T(?) | Basal-bark spraying, knapsack spraying |
| 584 | 9 | 00·07 | 2,4-D | Basal-bark spraying |

the study. Persons exposed to chlorophenols were excluded, with the exception of one patient and one control with exposure to both chemical groups. Table III gives latent period, duration of exposure and type of work for persons who had used phenoxyacetic acids. The relative risk of exposure to phenoxyacetic acids was calaculated as 5·3 (95% confidence interval, 2·4–11·5; Table IV).

The first report about soft-tissue sarcomas and previous exposure to phenoxyacetic acids was initiated by 3 patients admitted to the Department of Oncology in the autumn of 1976. These findings were followed by a pilot study which disclosed another 4 exposed patients. The relative risk was also calculated excluding the 3 "signal" cases and their controls, in order to check that the risk ratio obtained was not the result of pure chance. One of these patients was considered unexposed throughout the investigation since the next of kin did not confirm any exposure. The relative risk was then calculated as 4·7 (95% confidence interval 2·0–10·7; $\chi^2 = 13{\cdot}2$, $P < 0{\cdot}001$).

TABLE IV.—*Exposure to phenoxyacetic acids only. Patients and controls with exposure to chlorophenols are excluded*

| | Exp. | Non-exp. | Total |
|---|---|---|---|
| Case | 13 | 33 | 46 |
| Control | 14 | 187 | 201 |
| | 27 | 220 | 247 |

Relative risk: 5·3 (2·4–11·5).

$\chi^2 = 17{\cdot}4$.

$P < 0{\cdot}001$.

*Chlorophenols*

When analysing the effect of exposure to chlorophenols, patients and controls exposed to phenoxyacetic acids were excluded, except for one patient and one control with exposure to both chemical groups (Table VI). The calculation gave a

TABLE V.—*Exposure to chlorophenols only. Patients and controls exposed to phenoxyacetic acids are excluded*

| | Exp. | Non-exp. | Total |
|---|---|---|---|
| Case | 7 | 33 | 40 |
| Control | 6 | 187 | 193 |
| | 13 | 220 | 233 |

RR = 6·6.
$P < 0·001$.

relative risk of 6·6. Four patients (7·7%) and 14 controls (6·8%) reported the use of paints containing chlorophenols during their leisure. This implies that such use did not increase the risk for the studied disease. The exposure was in all cases occasional and at a low level. All chlorophenols and 2,4,5-T contain chlorinated dibenzodioxins as an impurity. Only 2 patients and 2 controls had been exposed to preparations not containing dioxins (2,4-D or MCPA) and obviously the observed risk ratios obtained for phenoxy acids and chlorophenols could equally have been caused by dioxins.

*Dichloro-diphenyl-trichloroethane (DDT)*

The use of DDT-treated plants was common during the 1950s and 1960s, and therefore the potential carcinogenic risk of such exposure was analysed. Two patients and 4 controls with unknown exposure were excluded. Four cases and 14 controls reported exposure to DDT, and the relative risk was then calculated as 1·2.

*Other exposures*

As there might be some other factor related to the occupations in which some people were exposed to the studied chemicals, the risk of the tumour of interest was also calculated for the unexposed persons in these occupations. Seven cases and 47 controls were assigned to that group. The relative risk was 0·6, which implied that the excess risk was related to the chemicals under consideration. Motorized sawing was studied in view of the exhaust fumes and their possible carcinogenic effect. No information was obtained from 2 patients and 2 controls, and they were consequently excluded. Eight cases and 41 controls reported exposure to exhaust fumes, and the relative risk was then calculated as 0·8. Emulsion agents such as diesel oil might have been present in the spraying aggregates. Information about such possible use was obtained from only 10 of the exposed persons. One patient reported such use during one week out of a total exposure of 34 weeks. One patient and 6 controls denied the use of diesel oil in an emulsion and 2 controls confirmed its use. No apparent difference in smoking habits between patients and controls was found. Fifty-one per cent of the patients were smokers (23% ex-smokers) compared with 48% of the controls (22% ex-smokers).

### DISCUSSION

The material consisted of 52 patients with soft-tissue sarcomas. All tumour histologies were reviewed by a pathologist. It is well known that the carcinogenic effect of a specific exposure is often most easily recognized in a rare type of tumour, and also that the aetiological connection in this situation is best documented by a case-control study. In a more common type of tumour, the effect may be blurred by a wider spectrum of exposures and, as regards the rarer tumours, cohort studies often contain too few cases for significant results. In this case a rare type of tumour acted as a signal, and the relation to the specific exposure was first suspected from clinical observations (Hardell, 1977). From a methodological point of view some aspects of the investigation need special comments.

The admission region of the Department of Oncology in Umeå includes the 3 most northern counties in Sweden. All soft-tissue sarcomas in this region are not examined at the department, but any selection of patients regarding possible exposure to phenoxyacetic acids or chlorophenols is unlikely. The probability of exposure to these chemicals is greater in rural areas than in urban areas, and to avoid bias the controls were matched for place of residence.

For dead patients, deceased controls were selected in order to achieve similar conditions when relatives of deceased patients and their matching controls were interviewed. Persons dying from a tumour disease were excluded from these controls, as possible carcinogenicity could be valid also for other types of tumours and thus blur the effect on the studied specific tumour type.

Only general information about the investigation was disclosed in the first telephone contact with the persons concerned and reference to phenoxyacetic acids or chlorophenols was avoided. The questionnaire included a large variety of questions in order to mask the purpose of the investigation. To minimize the risk of biasing the results, the person who made the supplementary interviews did not know whether the interviewed persons were patients or controls. The statements obtained from the questionnaires and the telephone interviews were considered as the single source of information about exposure. The next of kin of one of the first 3 observed patients did not report any exposure to phenoxyacetic acids. In this case the history in the record revealed massive exposure 15–4 years before the diagnosis. This patient was considered unexposed throughout the investigation in order to obtain a conservative estimate of the risk.

*Confounding*

The method described above for selecting controls excluded confounding factors such as sex, age, place of birth and year of death. Smoking habits were studied and showed no apparent difference between patients and control persons.

Motorized sawing work and related exhaust exposure was analysed, and no significant alteration of the risk could be shown. Theoretically, diesel oil might have been an uncontrolled confounding factor for the effect of phenoxyacetic acids, but hardly so for the chlorophenols. The information obtained was, however, insufficient for a proper evaluation. DDT might also be a confounding factor, but the calculation of relative risk showed no significant increase due to this chemical. The pure substances and impurities could not be evaluated separately as both phenoxyacetic acids and chlorophenols contained impurities such as chlorinated dioxins, dibenzofurans and carriers. Other pesticides might also constitute uncontrolled confounding factors. For example, stump painting with pikloram did occasionally occur during this period. No statements about exposure to pikloram or other such pesticides were obtained by the questionnaires.

*Conclusion*

The investigation showed an increased risk for soft-tissue sarcomas related to the use of phenoxyacetic acids or chlorophenols. It is most unlikely that the results were influenced by uncontrolled confounding factors or other defects in the validity of the study. A specific evaluation of the effect of separate chemical substances was not possible, as nearly all exposed persons were also exposed to chlorinated dioxins, including their most potent form, 2,3,7,8-tetrachlorodibenzo-p-dioxin (TCDD). The increased risk for this type of tumour after exposure to phenoxyacetic acids or chlorophenols can consequently be caused by the pure chemical substances, impurities in the commercial preparations or a combination of both.

This investigation was made possible by grants from the Swedish Work Environment Fund. The authors are especially grateful to Professor Lars-Gunnar Larsson, Umeå, and Professor Olav Axelson, Linköping, for valuable assistance in the methodological work. Professor Lennart Angervall, Göteborg, has reviewed all the histological specimens. Finally we would like to thank Lena Damber for her help with the interviews, and Monica Johnsson and Britta Lundgren for their editorial help.

### REFERENCES

AXELSON, O. & SUNDELL, L. (1974) Herbicide exposure, mortality and tumor incidence. An epidemiological investigation on Swedish railroad workers. *Work-Environ. Hlth*, **11**, 21.

AXELSON, O. & SUNDELL, L. (1977) (In Swedish) Correspondence: Phenoxyacetic acids and cancer. *Läkartidningen*, **74**, 2887.

HARDELL, L. (1977) (In Swedish) Soft tissue sarcomas and exposure to phenoxyacetic acids—a clinical observation. *Läkartidningen*, **74**, 2753.

LEVIN, J. O., RAPPE, C. & NILSSON, C. A. (1976) Use of chlorophenols as fungicides in sawmills. *Scand. J. Work Environ. & Health*, **2**, 71.

MIETTINEN, O. (1970) Estimation of relative risk from individually matched series. *Biometrics*, **26**, **75**.

MIETTINEN, O. (1972) Components of the crude risk ratio. *Am. J. Epidemiology*, **96**, 168.

MIETTINEN, O. (1976) Estimability and estimation in case-referent studies. *Am. J. Epidemiology*, **103**, 226.

MURANYI-KOVACS, I., RUDALI, G. & IMBERT, J. (1976) Bioassay of 2,4,5-trichlorophenoxyacetic acid for carcinogenicity in mice. *Br. J. Cancer*, **33**, 626.

THIESS, A. M. & FRENTZEL-BEYME, R. (1978) Mortality study of persons exposed to dioxin after an accident which occurred in the BASF on 13th November 1953. Paper presented at the *Working Group of the NIEHS and the IARC*, Lyon, January 1978.

TÓTH, K., SUGÁR, J., SOMFAI-RELLE, S. & BENCE, J. (1978) Carcinogenic bioassay of the herbicide 2,4,5-trichlorophenoxy-ethanol (TCPE) with different 2,3,7,8-tetrachlorodibenzo-p-dioxin (dioxin) content in Swiss mice. *Prog. Biochem. Pharmacol.*, **14**, 82.


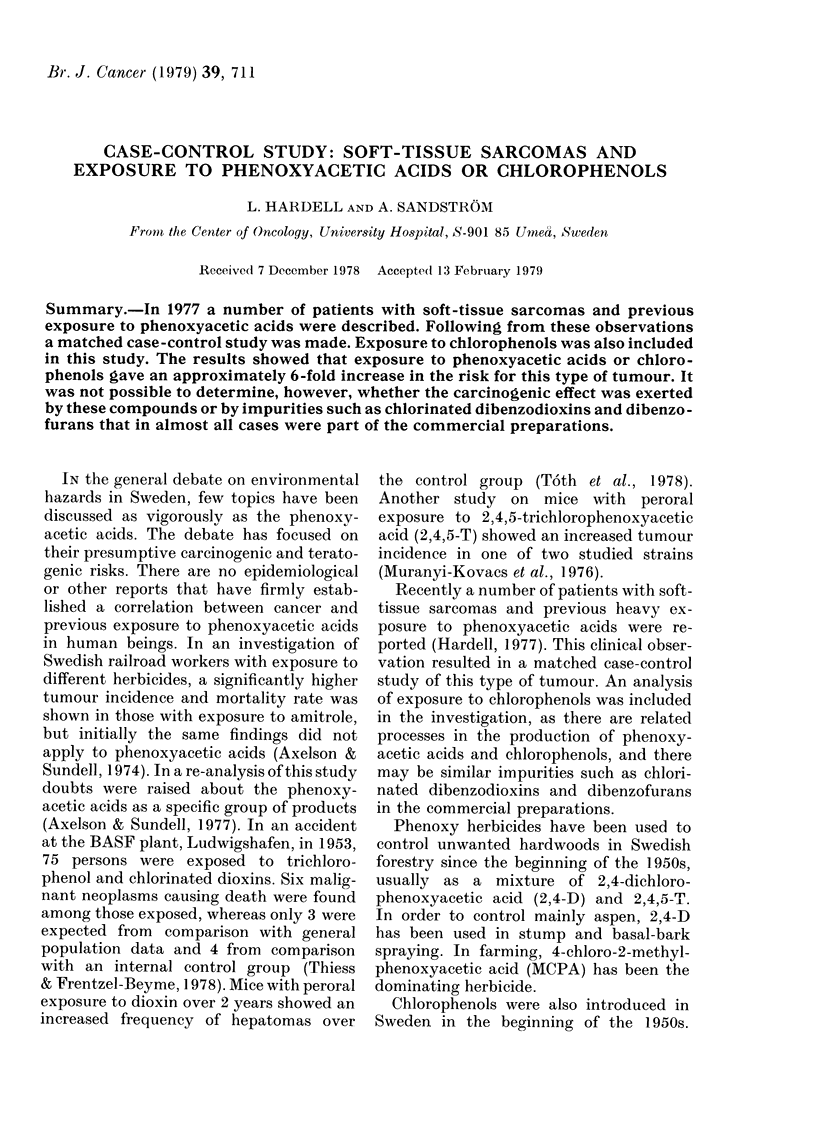

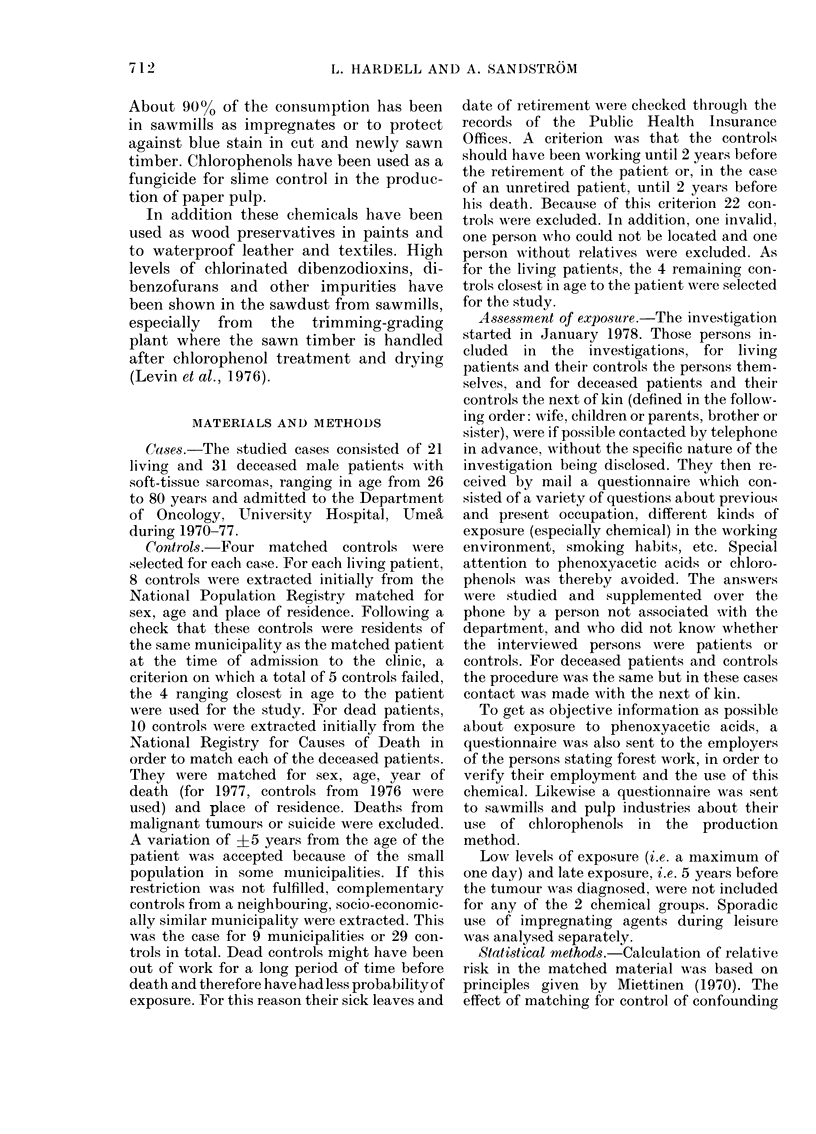

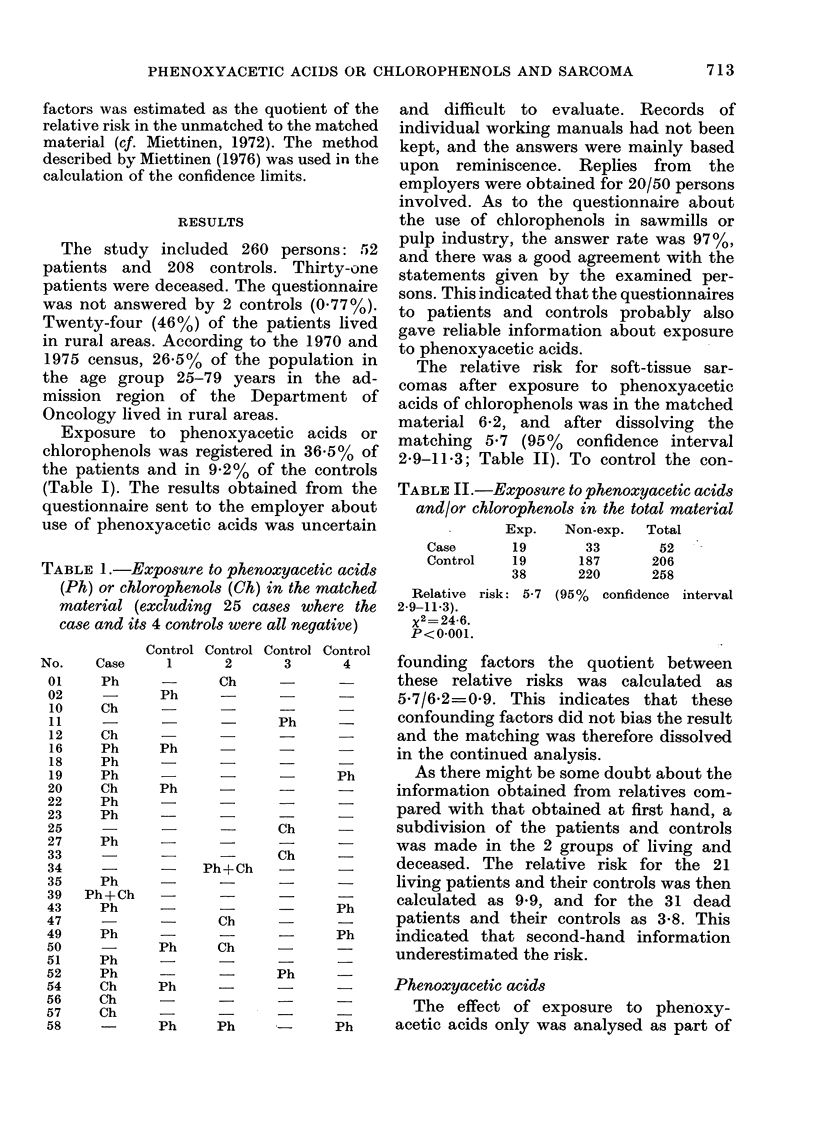

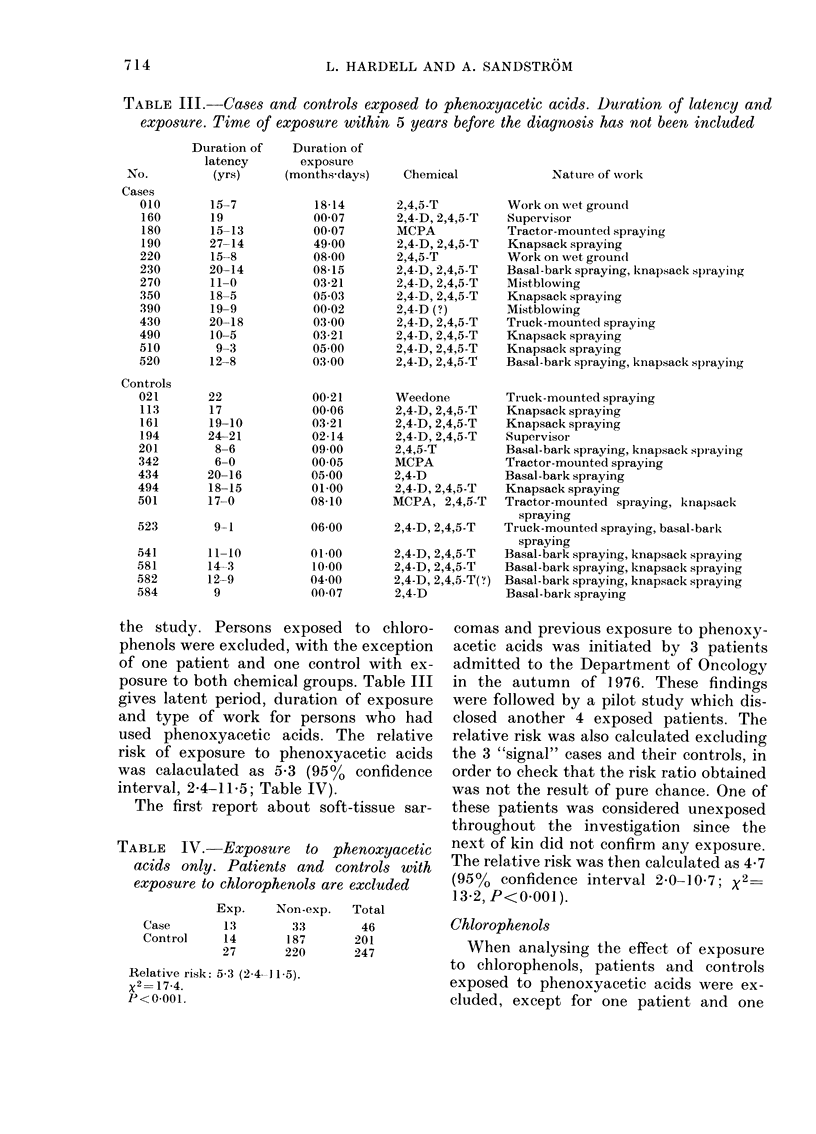

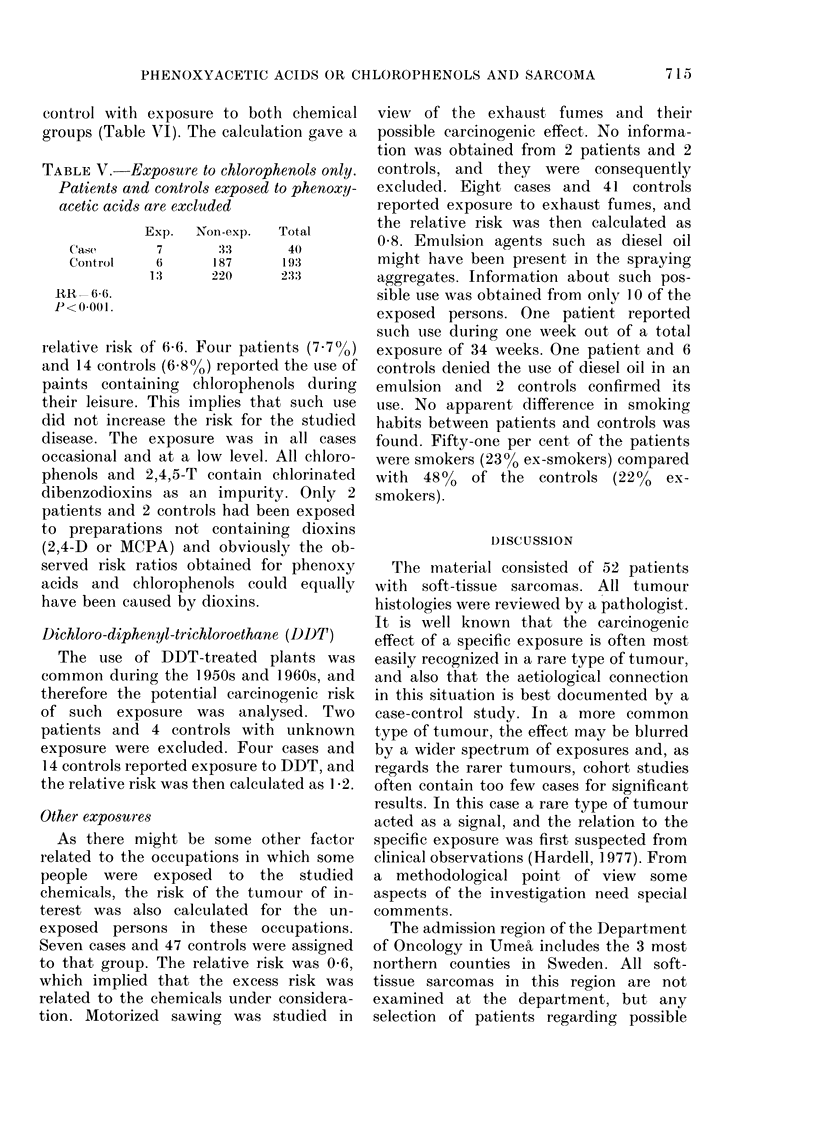

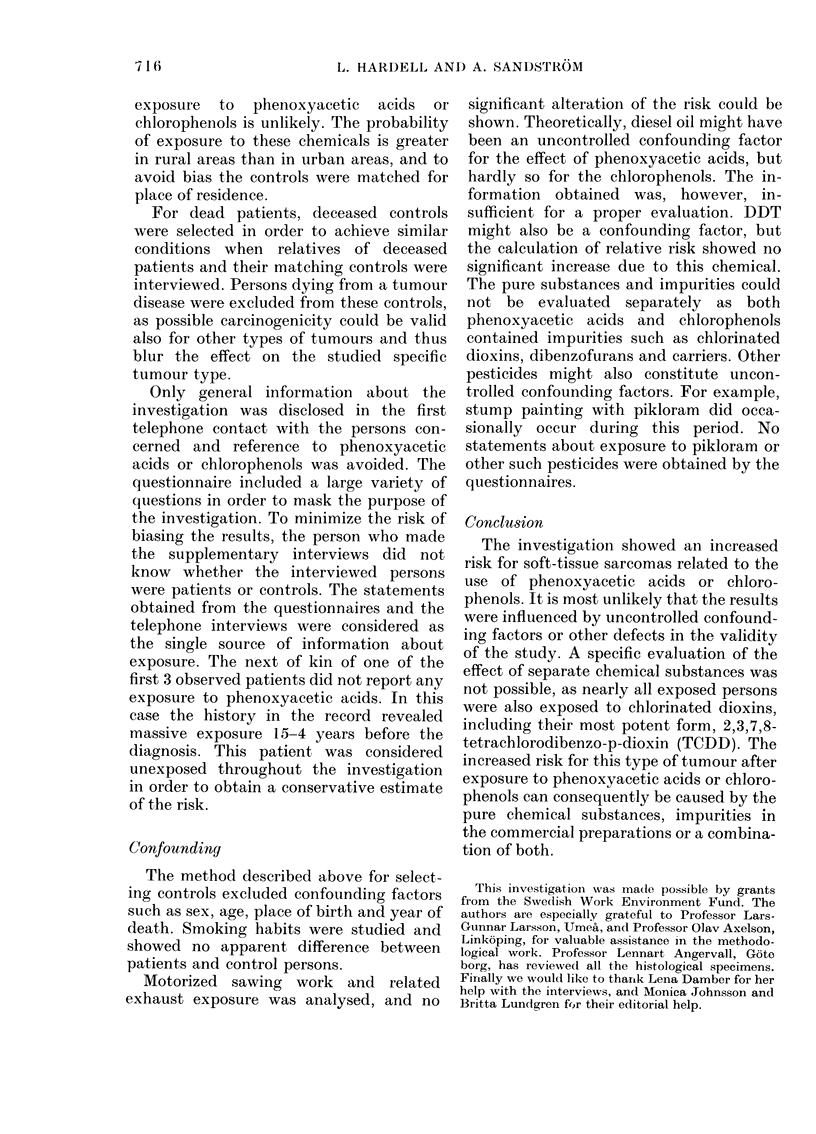

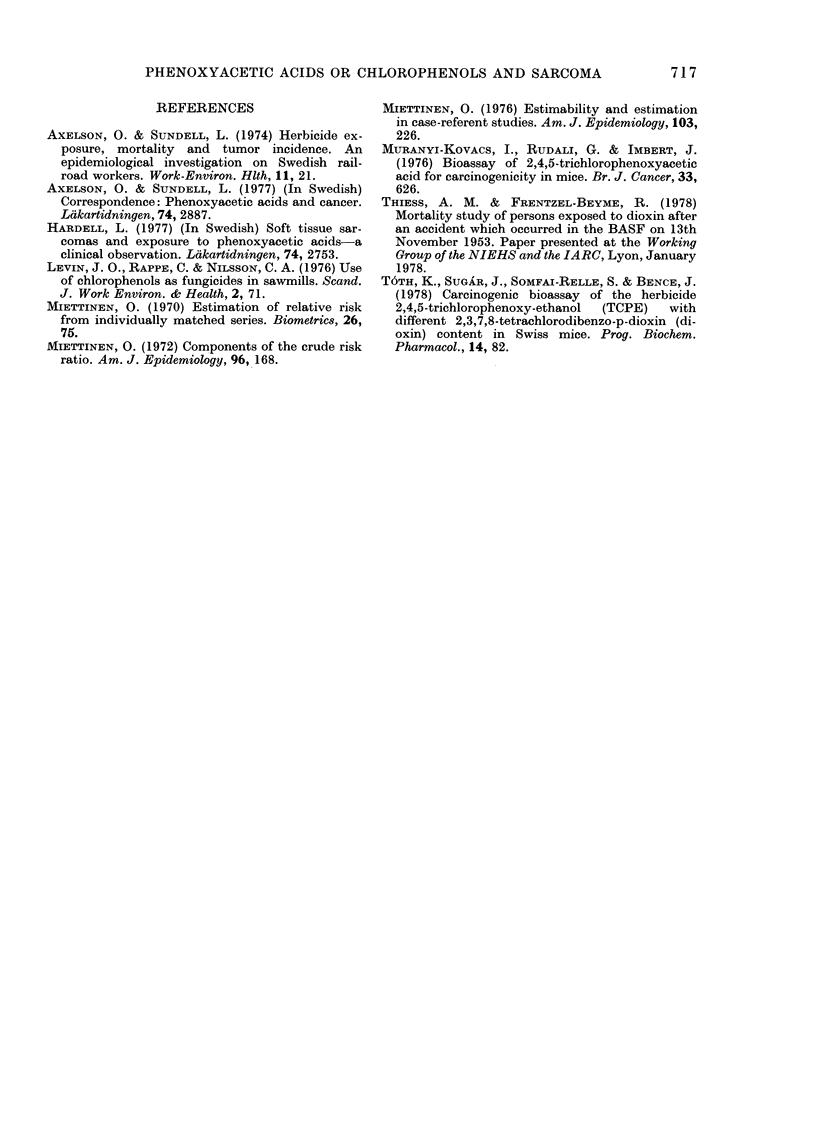


## References

[OCR_00920] Axelson O., Sundell L. (1974). Herbicide exposure, mortality and tumor incidence. An epidemiological investigation on Swedish railroad workers.. Work Environ Health.

[OCR_00936] Levin J. O., Rappe C., Nilsson C. A. (1976). Use of chlorophenols as fungicides in sawmills.. Scand J Work Environ Health.

[OCR_00946] Miettinen O. S. (1972). Components of the crude risk ratio.. Am J Epidemiol.

[OCR_00941] Miettinen O. S. (1970). Estimation of relative risk from individually matched series.. Biometrics.

[OCR_00950] Miettinen O. (1976). Estimability and estimation in case-referent studies.. Am J Epidemiol.

[OCR_00955] Muranyi-Kovacs I., Rudali G., Imbert J. (1976). Bioassay of 2, 4, 5-trichlorophenoxyacetic acid for carcinogenicity in mice.. Br J Cancer.

